# Preoperative prognostic nutritional index value as a predictive factor for postoperative delirium in older adult patients with hip fractures: a secondary analysis

**DOI:** 10.1186/s12877-023-04629-z

**Published:** 2024-01-04

**Authors:** Xinning Mi, Yunyang Jia, Yanan Song, Kaixi Liu, Taotao Liu, Dengyang Han, Ning Yang, Geng Wang, Xiangyang Guo, Yi Yuan, Zhengqian Li

**Affiliations:** 1 Department of Anesthesiology, Peking University Third Hospital, Beijing, 100191 China; 2 Department of Orthopaedics & Traumatology, Beijing Jishuitan Hospital, Capital Medical University, Beijing, 100035 China; 3 Department of Anesthesiology, Beijing Jishuitan Hospital, Capital Medical University, Beijing, 100035 China; 4 Beijing Center of Quality Control and Improvement On Clinical Anesthesia, Beijing, 100191 China; 5 Perioperative Medicine Branch of China International Exchange and Promotive Association for Medical and Health Care (CPAM), Beijing, 100191 China

**Keywords:** Nutritional status, Prognostic nutritional index, Postoperative delirium, Hip fracture, Older adult patients

## Abstract

**Background:**

Malnutrition is a common geriatric syndrome and can be targeted preoperatively to decrease the risk of postoperative delirium (POD) in older adult patients. To analyze the value of the prognostic nutritional index (PNI) to predict the incidence of POD in older adult patients with hip fractures.

**Methods:**

This was a prospective, observational, cohort study of older adult patients with hip fractures. Preoperative PNI was calculated as 10 × serum albumin (g/dL) + 0.005 × total lymphocyte count (/μL) using preoperative laboratory results. Patients were divided into POD and non-POD groups using the Confusion Assessment Method (CAM). The risk factors associated with POD as well as the relationship between PNI values and the incidence of POD were analyzed using univariate and multivariate logistic regression analyses. The predictive value of PNI for POD was assessed using receiver operating characteristic curve analysis.

**Results:**

In this cohort of 369 patients who underwent hip fracture surgery, 67 patients (18.2%) were diagnosed with POD by the CAM results. Low PNI increased the risk of POD (odds ratio (OR) = 0.928, 95% confidence interval (CI): 0.864–0.997). General anesthesia (OR = 2.307, 95% CI: 1.279–4.162) and Mini-Mental State Examination (MMSE) score (OR = 0.956, 95% CI: 0.920–0.994) were also identified as risk factors for POD. Receiver operating characteristic curve analysis suggested that PNI combined with the anesthetic method and MMSE score may be used as a potential predictive indicator of POD after hip fracture surgery.

**Conclusion:**

Preoperative PNI value is related to POD in older adult patients with hip fractures.

**Trial registration:**

This secondary analysis study was approved by the Peking University Third Hospital Medical Science Research Ethics Committee (approval No. M2022578) and registered in the Chinese Clinical Trial Registry (ChiCTR2300070569).

## Background

Postoperative delirium (POD) is an acute neuropsychiatric syndrome characterized by inattention, fluctuating levels of consciousness, and/or disorganized thinking, which is associated with increased morbidity, mortality, and health care costs [1, 2]. As anesthetic and surgical care continues to improve, a significantly greater number of older adult patients are undergoing elective surgical procedures. However, these procedures are associated with an increased POD risk perioperatively. It is of great importance to perform interventions for older adult patients preoperatively to optimize perioperative management and prevent POD.

Age, malnutrition, perioperative low albumin, pre- and postoperative pain, blood transfusions, and intraoperative hypotension are risk factors for POD [3, 4]. Malnutrition is a common geriatric syndrome and can be recognized and targeted preoperatively to decrease the risk of POD in older adult patients. The presence of malnutrition is determined using a series of scales, namely, the Mini-Nutritional Assessment, Mini-Nutritional Assessment Short Form, Nutrition Risk Screening 2002, and several laboratory parameters [5]. However, assessment scales have limitations. For example, completing questionnaires may be time-consuming and complex for older adult patients, and subjective errors are inevitable. Instead, laboratory parameters, such as serum albumin and total lymphocyte count, which reflect a patient’s nutritional status prior to surgery, are more objective than assessment scales, and the results are easier to obtain in surgical patients [6–9].

Recently, the prognostic nutritional index (PNI) has been popularized, which is calculated as 10 × serum albumin (g/dL) + 0.005 × total lymphocyte count (/μL). Low PNI is a significant risk factor for POD in older adult patients with colorectal cancer and patients who undergo adult spinal deformity surgeries [10, 11]. Unfortunately, few studies have evaluated the association between preoperative nutritional status and the incidence of POD in older adult patients with hip fractures who undergo surgical repair. These patients are more susceptible to preoperative malnutrition and POD compared with other patient groups [12]. However, the effectiveness of PNI as a dependable predictive index for POD must be verified.

In the present study, we hypothesized that in older adult patients who undergo surgical repair for hip fractures, low PNI is associated with the incidence of POD. The study aimed to prove that preoperative nutritional status can be objectively and easily predicted to identify patients susceptible to POD.

## Methods

This was a secondary analysis based on two prospective, observational, single-center, cohort studies performed in the Geriatric Orthopedics Unit of Beijing Jishuitan Hospital from June 2020 to March 2022. The primary objective of the study was to explore the association between PNI and the incidence of POD in older adult patients with hip fractures. Accordingly, we analyzed PNI as a predictive factor of POD in older adult patients who underwent surgical repair for hip fractures. This study aimed to identify patients at high risk of developing POD using PNI, and to administer preoperative nutritional support as early as possible, to prevent POD.

### Ethics approval and clinical registration

This secondary analysis study was approved by the Peking University Third Hospital Medical Science Research Ethics Committee (approval No. M2022578) and registered in the Chinese Clinical Trial Registry (ChiCTR2300070569). The original two prospective, observational, single-center, cohort studies were approved by the Medical Science Research Ethics Committees of Beijing Jishuitan Hospital (JLKS201901-04, JLKS202009-10, respectively) and registered in the Chinese Clinical Trial Registry (ChiCTR1900027393, ChiCTR2000038924, respectively). All patients provided informed consent for the collection and analysis of their clinical data and serum samples.

### Patients

This study was a secondary analysis performed at Beijing Jishuitan Hospital between 1 June 2020 and 30 March 2022. According to the previous study [13], the inclusion criteria were: age ≥ 65 years, hospital admission for surgical treatment of hip fracture, and American Society of Anesthesiologists physical status classification I–III. The exclusion criteria were: central nervous system disorders already existed preoperatively or with a previous history of such diseases, including preoperative delirium, Parkinson’s disease, dementia (including dementia due to Parkinson’s disease, Alzheimer’s disease, and Lewy body dementia), stroke within the previous 6 months or other central nervous system disorders; other factors that might affect the incidence of POD including multiple traumas and transfer to the intensive care unit postoperatively; communication difficulties and severe hearing or vision impairment which were unable to finish assessment scales, and unwillingness to participate in the study or unexpected discharge.

### Perioperative clinical assessment

Nutritional status was assessed via PNI, which was calculated as 10 × serum albumin (g/dL) + 0.005 × total lymphocyte count (/μL). Serum albumin and total lymphocyte count were obtained from preoperative laboratory results.

All participants were interviewed the day before surgery, and the following baseline data were collected: demographic information, laboratory results, American Society of Anesthesiologists physical status, age-adjusted Charlson comorbidity index [14], Mini-Mental State Examination (MMSE) [15], education level, and Activities of Daily Living [16]. Pain intensity was assessed using a numerical rating scale [17]. Other information namely comorbidities, medical history, and fracture classification was collected from the patients’ medical records. The laboratory results were collected from the preoperative examinations processed 2 ~ 5 days before surgery. History collection and physical evaluation were performed by trained investigators.

### Diagnosis of POD

The Confusion Assessment Method (CAM) was used to exclude patients with preoperative delirium and to diagnose POD, as previously described [13, 18]. Four items (acute onset and fluctuating course, inattention, disorganized thinking, altered level of consciousness) constitute the CAM. When diagnosing delirium, it is important to obtain information from a reliable source about the patient's acute onset and fluctuating course. Inattention may be indicated by difficulty focusing attention, while disorganized thinking may be shown by incoherent thoughts. The patient's level of consciousness may be altered, categorized as consciousness, vigilant, lethargic, stupor, or coma. To diagnose delirium using the CAM method, the first two items must be present along with either of the last two items. All participants were followed for the first 2 postoperative days, a period in which POD is usually diagnosed after hip fracture surgery in older adult patients. Cognitive assessment was performed through twice-daily visits and by a fixed geriatric-care physician.

### Anesthesia and analgesia

No sedatives and/or anticholinergic drugs were administered to any patients as adjuvant medications before anesthesia. The anesthesia method (general anesthesia or spinal anesthesia) was determined by anesthesiologists in communication with the patients. After entering the operation room, patients received inhalational mask oxygen at 6 L/min. Heart rate, electrocardiography, and pulse oximetry were monitored. Radial artery catheterization was performed to directly measure mean arterial pressure and for blood gas analysis. All patients received an ultrasound-guided iliac fascia block and were given 30 mL of 0.33% ropivacaine for regional anesthesia and postoperative analgesia. Patients who received spinal anesthesia underwent single-dose subarachnoid spinal anesthesia as an injection of 8–10 mg of 0.3% ropivacaine at the L2–3 or L3–4 levels, and the anesthesia plane reached T10. Patients who received general anesthesia underwent intravenous induction with propofol and fentanyl. Rocuronium or cisatracurium was administered intravenously when the patient lost consciousness. Endotracheal intubation or laryngeal mask placement was performed 2 min later. Sevoflurane, remifentanil, rocuronium, or cisatracurium were used for anesthetic maintenance. The end-tidal partial pressure of carbon dioxide and bispectral index value were monitored intraoperatively in patients who underwent general anesthesia. During the operation, mean arterial pressure and heart rate were maintained within 20% of the baseline values, partial pressure end-tidal carbon dioxide was maintained at 30–35 mmHg, and the bispectral index value was maintained at 40–60 for patients who underwent general anesthesia.

For postoperative analgesia, all patients received intravenous patient-controlled analgesia with 1.5 μg/kg fentanyl, 200 mg flurbiprofen axetil, and 10 mg tropisetron hydrochloride in 100 ml saline in addition to the preoperative iliac fascia block.

### Sample size calculation

An observational study conducted in China showed that patients with POD had a PNI of 39.4 ± 10.2, whereas those without POD had a PNI of 47.4 ± 11.7 [19]. Based on the calculated PNI mean, the study estimated that both the POD and non-POD groups needed to recruit 29 effective cases to achieve an 80% power at an α value of 0.05. To control for confounding factors such as age, hip fracture type, ASA classification, duration of surgery, intraoperative bleeding, etc., it was proposed to use logistic regression analysis. With an Events Per Variable equal to 5, 25 patients would be required in the POD group. As the incidence of POD in our previous study was 18.9%, the total sample size needed for the study was calculated to be 132 participants. To account for a potential dropout rate of 10%, the study aimed to recruit 147 participants.

### Statistical analysis

The Kolmogorov–Smirnov test was used to analyze the distribution of quantitative variables. Measurement data that followed a normal distribution were presented as mean ± standard deviation, and the independent sample’s *t*-test was used for analysis. Measurement data that did not follow a normal distribution were expressed as median (minimum, maximum), and the rank sum test was used for analysis. The chi-square test was used to analyze the enumeration data. The patients were divided into a POD group and a non-POD group based on the CAM results. Univariate analysis was used to screen for differences in POD-associated factors between the groups, and binary logistic regression was used to identify the risk factors for POD. The areas under the receiver operating characteristic curve (AUCs) of the risk factors were then calculated. All data were statistically analyzed using SPSS 25.0 (IBM Corp., Armonk, NY, USA), and *P* < 0.05 was considered statistically significant.

## Results

### Baseline characteristics

**Fig. 1:**
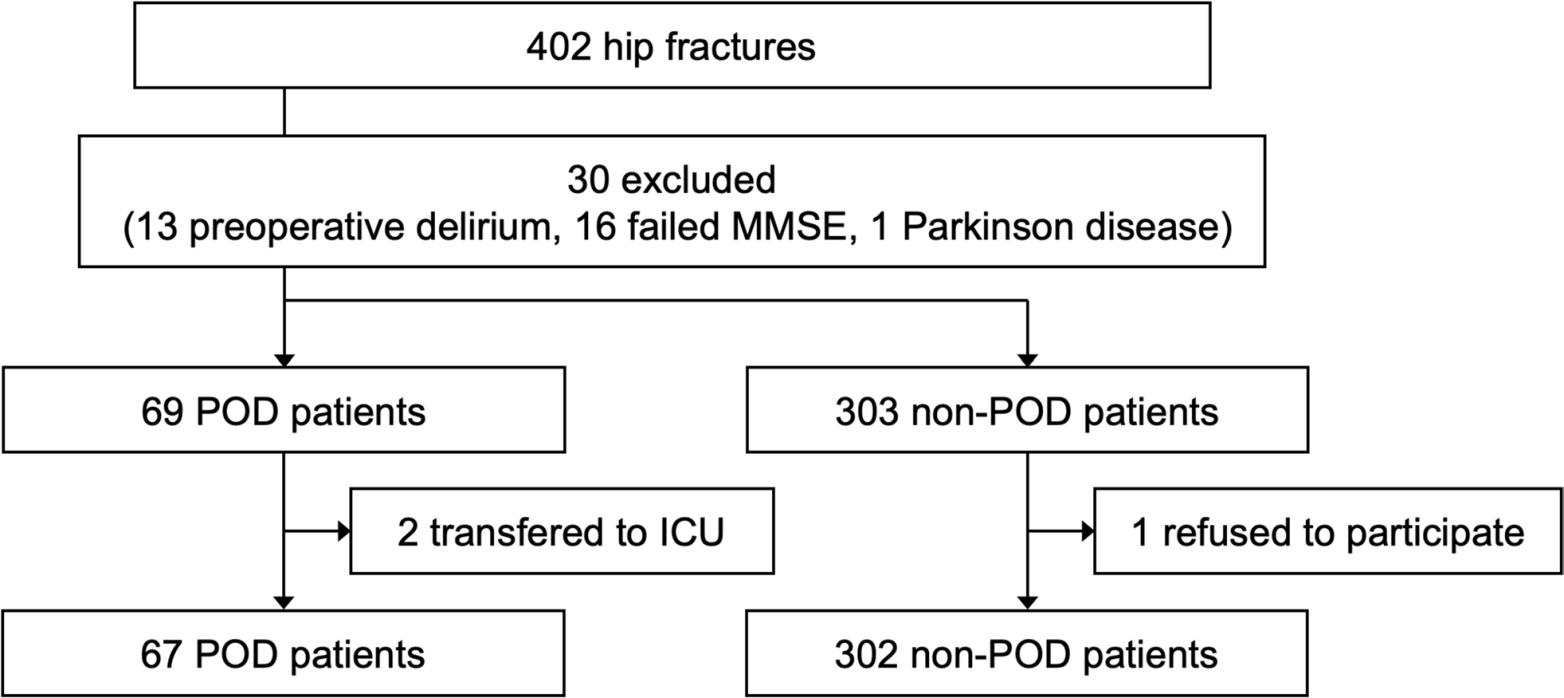
Flowchart of the research design. A total of 369 older adult patients with hip fractures were included in this study. MMSE, Mini-Mental State Examination; POD, postoperative delirium; ICU, intensive care unit

A total of 369 older adult patients with hip fractures were included in this study. Of these, 67 patients were diagnosed with POD on the basis of the CAM results (Fig. 1). Overall baseline and clinical characteristics and the majority of the intraoperative characteristics were well-balanced between the POD and non-POD groups except for the comorbidity of hypertension, MMSE score, and anesthesia method (Table 1). Specifically, hypertension, low MMSE, and general anesthesia were risk factors for POD (*p* < 0.05).

**Table 1:** Comparison of baseline characteristics and other covariables between patients with and without postoperative delirium

	Non-POD (*n* = 302)	POD (*n* = 67)	*p*-value	Standardized difference
Sex, male	221 (73.2%)	44 (65.7%)	0.217	0.158
Age, yr	78.54 ± 6.78	79.85 ± 7.32	0.16	0.179
Weight, kg	61.27 ± 11.09	61.69 ± 10.92	0.782	0.038
BMI, kg/m^2^	23.45 ± 3.62	23.79 ± 3.61	0.485	0.095
ASA physical status class			0.595	0.096
I	5 (1.66%)	0 (0%)		
II	194 (64.24%)	41 (61.19%)		
III	103 (34.11%)	26 (38.81%)		
ACCI	3 (2,4)	4 (2.2,4)	0.116	0.230
Hypertension	179 (59.3%)	49 (73.1%)	0.035	0.313
Diabetes	85 (28.1%)	25 (37.3%)	0.138	0.190
Ischemic heart disease	71 (23.5%)	18 (26.9%)	0.561	0.076
Stroke	65 (21.5%)	11 (16.4%)	0.35	0.138
Chronic obstructive pulmonary disease	33 (11.0%)	7 (10.4%)	0.902	0.017
Smoking status, yes	59 (19.5%)	16 (23.9%)	0.424	0.102
Alcohol abuse, yes	27 (8.9%)	10 (14.9%)	0.140	0.168
ADL, points	14 (14,15)	14 (14,16)	0.122	0.236
Education, yr	9 (4,12)	9 (6,12)	0.683	0.054
MMSE, points	26 (22,28)	24 (18,27.5)	0.009	0.327
Anesthesia method			0.004	0.390
Spinal anesthesia	189 (62.6%)	29 (43.3%)		
General anesthesia	113 (37.4%)	38 (56.7%)		
Duration of anesthesia, min	90 (70,110)	90 (75,100)	0.886	0.109
Duration of surgery, min	60 (40,80)	60 (30,75)	0.387	0.243
Time from injury to surgery, hours	96 (70,124.6)	81.8 (63.8,144.9)	0.492	0.143
Type of fracture			0.641	0.008
Intertrochanteric	152 (50.3%)	34 (50.7%)		
Femoral neck	148 (49%)	32 (47.8%)		
Subtrochanteric	2 (0.7%)	1 (1.5%)		
Hospital stay, days	4 (3,5)	5 (3,5)	0.621	0.093

Categorical variables are expressed as n (%). Data with a normal distribution are presented as mean ± standard deviation, whereas non-normally distributed data are presented as median (25th percentile, 75th percentile). *POD* postoperative delirium, *BMI* body mass index, *ASA* American Society of Anesthesiologists, *ACCI* age-adjusted Charlson comorbidity index, *ADL* activities of daily living, *MMSE* mini-mental state examination

### Preoperative nutritional status and laboratory results

Patients with low PNI, low serum albumin, and low thyroid stimulating hormone concentrations, were more likely to be diagnosed with POD compared with those without POD. Since the distribution of the PNI data is non-normal, we pre-suppose the median PNI of 45.6 as the cut-off value. We then divided PNI into low and high PNI by the cut-off value of 45.6, and we found that PNI < 45.6 could be used as a predictive factor for the incidence of POD. In contrast, lymphocyte count and other laboratory results showed no significant differences between POD and non-POD patients (Table 2).

**Table 2:** Comparison of nutritional and laboratory results between patients with and without postoperative delirium

	Non-POD (*n* = 302)	POD (*n* = 67)	*p*-value
PNI	46.8 (44.2,49.3)	45.6 (43.5,47.8)	0.019
PNI			0.039
< 45.6	122 (41.36%)	37 (55.22%)	
≥ 45.6	173 (58.64%)	30 (44.78%)	
Albumin, g/L	41.3 (39.2,43.2)	40.6 (38.6,41.8)	0.028
Lymphocyte, × 10^9^/L	1.1 (0.8,1.4)	1.1 (0.8,1.5)	0.619
Leukocyte, × 10^9^/L	9.7 (7.9,11.7)	9.6 (8.1,11.5)	0.827
Erythrocyte, × 10^12^/L	4 (3.6,4.3)	3.9 (3.6,4.2)	0.827
Hemoglobin, g/L	121 (110,130)	119.5 (107,127.5)	0.361
Platelet, × 10^9^/L	198.5 (161.2,238.8)	205 (174,257)	0.246
Sodium, mmol/L	138 (136,140)	138 (136.2,140)	0.831
Chlorine, mmol/L	103 (100,105.2)	102 (99.2,105)	0.12
Potassium, mmol/L	3.9 (3.7,4.2)	3.9 (3.6,4.2)	0.496
Calcium, mmol/L	2.2 (2.1,2.3)	2.2 (2.1,2.3)	0.22
ALT, IU/L	14 (11,17)	12 (10,16)	0.063
AST, IU/L	18 (16,22)	18 (15,22)	0.425
Urea, mmol/L	6.5 (5.3,8.3)	7.4 (5.7,9.4)	0.061
Creatine, μmol/L	57 (47.8,70)	59.5 (52.2,75.8)	0.107
Total protein, g/L	65.9 (62,70.6)	65.7 (62,68.2)	0.386
Glucose, mmol/L	7.7 (6.6,10.1)	7.7 (6.6,9)	0.448
Glycosylated hemoglobin, %	5.8 (5.4,6.4)	6 (5.5,6.6)	0.239
PaCO_2_, mmHg	32.5 (29.4,36.1)	32.2 (29.5,34.6)	0.774
PaO_2_, mmHg	73.7 (66.8,81.4)	72.3 (66.9,78.9)	0.252
Lactic acid, mmol/L	1.1 (0.8,1.6)	0.9 (0.7,1.4)	0.472
C-reactive protein, mg/L	18 (4.6,58.1)	39.6 (6.9,65.6)	0.219
Thyroid stimulating hormone, mIU/L	1.8 (1,3.2)	1.3 (0.7,1.9)	0.003
Total tetraiodothyroxine, ng/ml	97.4 (83.4,112.9)	100.2 (86.4,113.6)	0.329
Total triiodothyroxine, ng/ml	1.2 (1.1,1.6)	1.3 (1.1,1.6)	0.939
Free tetraiodothyroxine, ng/ml	16.5 (14.4,18.7)	16.1 (14.4,18.8)	0.914
Free triiodothyroxine, ng/ml	3.3 (2.9,3.8)	3.1 (2.7,3.5)	0.054

Non-normally distributed data are presented as median (25th percentile, 75th percentile). *POD* postoperative delirium, *PNI* prognostic nutritional index, *ALT* alanine aminotransferase, *AST* aspartate aminotransferase, *PaCO*_*2*_ partial pressure of arterial carbon dioxide, *PaO*_*2*_ partial pressure of arterial oxygen

### Multiple logistic regression of the predisposing factors for POD

After adjusting for confounders, binary logistic regression showed that the significant preoperative risk factors for POD after hip fracture surgery were PNI [adjusted odds ratio (OR) = 0.928, 95% confidence interval (CI): (0.864, 0.997)], general anesthesia [(adjusted OR = 2.307, 95% CI: (1.279, 4.162)), and MMSE score [adjusted OR = 0.956, 95% CI: (0.920, 0.994)] (Table 3).

**Table 3:** Multiple logistic regression analysis of the predisposing factors for postoperative delirium

	Adjusted OR	95% CI	*p*-value
PNI	0.928	(0.864, 0.997)	0.040
Anesthesia method	2.307	(1.279, 4.162)	0.005
MMSE	0.956	(0.920, 0.994)	0.022
Hypertension	1.709	(0.902, 3.237)	0.100
Thyroid stimulating hormone, mIU/L	0.944	(0.842, 1.058)	0.323

*OR* odds ratio, *CI* confidence interval, *PNI* prognostic nutritional index, *MMSE* mini-mental state examination.

### Evaluation of PNI as a potential prognostic marker for POD

**Fig. 2:**
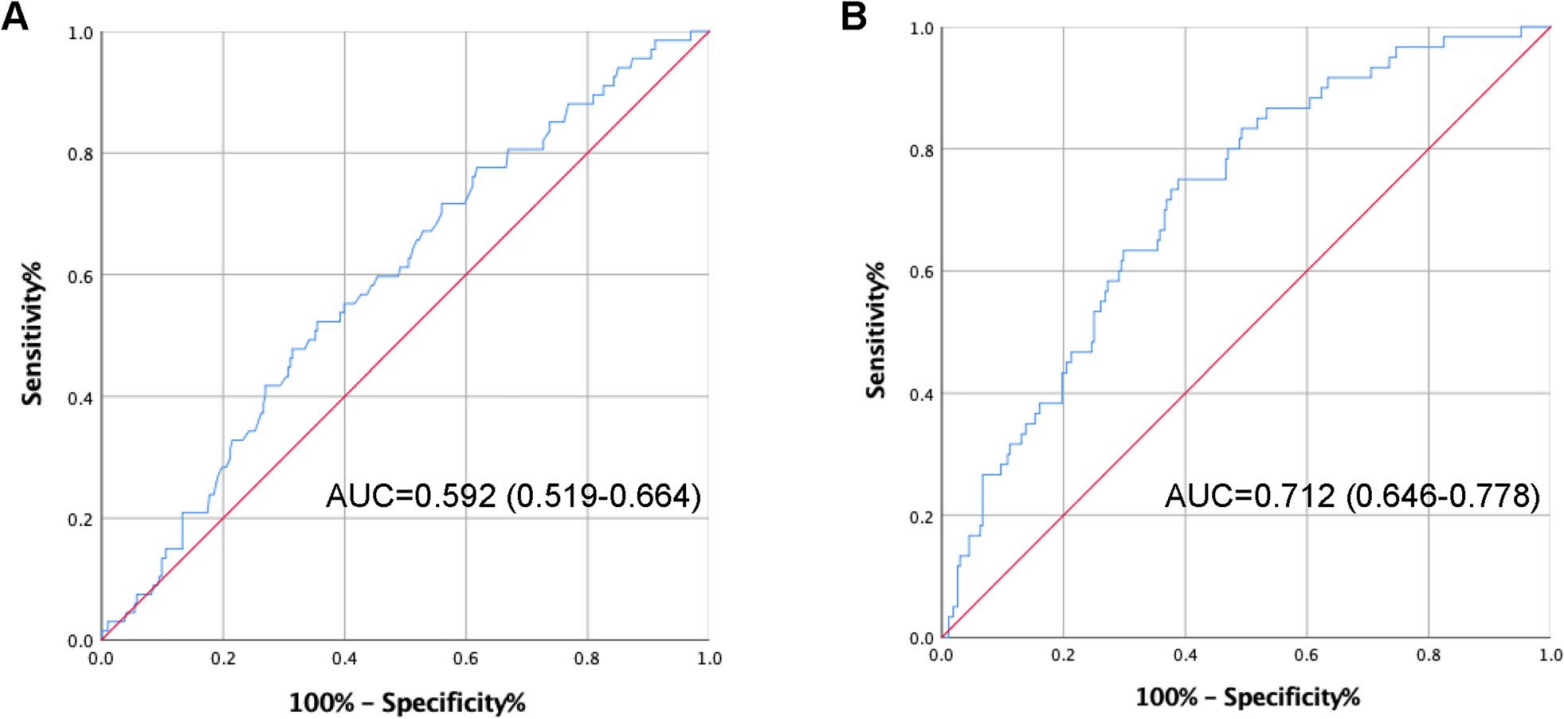
Receiver operating characteristic curve analysis of the prognostic nutritional index (PNI) and other risk factors for postoperative delirium; (**A**) results of PNI alone; (**B**) result of PNI combined with the anesthetic method and Mini-Mental State Examination score. AUC, area under the curve

The risk factors associated with POD shown in Table 3 were examined by receiver operating characteristic curve (ROC) analyses, and the AUC value for PNI was 0.592 [95% CI: (0.519, 0.664), sensitivity = 52.2%, specificity = 64.5%] (Fig. 2A). According to Youden index, the optimal cut-off values were verified as 45.675 for PNI. After combining general anesthesia and MMSE score, the AUC was 0.712 [95% CI: (0.646, 0.778), sensitivity = 75.0%, specificity = 61.2%] with a median predictive accuracy (Fig. 2B), suggesting that PNI combined with the anesthetic method and MMSE score rather than PNI alone may be used as a potential predictive indicator of POD after hip fracture surgery.

## Discussion

Although PNI is a convenient, objective, and easy-to-perform indicator to estimate a patient’s nutritional status, its effectiveness as a dependable predictive index for POD must be verified. In this secondary analysis of two prospective cohort studies, we evaluated PNI as a prognostic tool in older adult patients who underwent hip fracture surgery and found that low preoperative PNI was associated with an increased risk of POD compared with high preoperative PNI. Preoperative PNI combined with other risk factors, namely general anesthesia and a low MMSE score, could be used as a median-accurate comprehensive prognostic method for predicting POD, with an AUC value of 0.712 compared to preoperative PNI alone with an AUC value of 0.592.

PNI was first advanced in 1980 by Buzby et al*.* as a quantitative evaluation indicator to analyze the association between the risk of postoperative complications and baseline nutritional status [20]. In 1984, Onodera et al*.* revised the calculation of PNI as the present formula [21]. PNI has been used as a prognostic predictor in cancer patients [22, 23] and recently, has also been used to predict the incidence of POD in surgical patients because frailty and malnutrition are associated with POD [24]. Several studies have evaluated PNI as a useful factor for predicting POD after noncardiac surgery [25–27]. Additionally, one study found that older adult patients with hip fractures tended to present with an inadequate nutrient intake for their requirements and had a higher prevalence of malnutrition compared with younger patients [12]. Hence, we evaluated older adult patients with hip fractures in the present study.

The etiology of POD remains unclear. Our previous prospective cohort clinical trial found that the anesthetic method was associated with the incidence of POD, and patients diagnosed with POD were more likely to have lower MMSE scores compared with those without POD [13], which was consistent with the results in the present study. In the present study, the anesthetic method and MMSE score combined with PNI was a stronger predictive method of the incidence of POD compared with PNI alone. However, the MMSE score is an uncorrectable factor preoperatively, while PNI can be corrected easily in a short period before surgery and as a long-term lifestyle improvement in older adult patients. A meta-analysis showed that oral nutritional intervention before surgery could increase serum total protein concentration and decrease the incidence of postoperative complications [28]. A clinical trial found that perioperative taurine supplementation in older adult patients with hip fractures attenuated postoperative oxidative stress [29], as a potential mechanism of POD [30, 31]. However, the efficacy of preoperative nutritional intervention on PNI and the incidence of POD must be studied further.

PNI is often associated with systemic immune-inflammatory response index (SII) to assess the prognosis of cancer patients [32, 33], suggesting PNI is not only a nutritional index but also an inflammation-related marker. On one hand, hypoalbuminemia is associated with postoperative infection and inflammation. Specifically, inflammation increases capillary permeability and the escape of serum albumin, leading to hypoalbuminemia. While hypoalbuminemia is also an independent risk factor of postoperative infection [34]. On the other hand, lymphocytes are an important component of the immune process, low lymphocyte count is used as a marker of inflammation and immunosuppression [35, 36]. With these considerations, PNI is a comprehensive predictor of POD, which is consistent with findings in animal studies demonstrating that systematic inflammation plays a role in the mechanism of POD [37, 38]. Further exploration is needed to determine the precise association between low PNI and neuroinflammation rather than systemic inflammation.

The major strength of our study is that we demonstrated PNI as a prognostic predictor of POD in older adult patients with hip fractures. Through preoperative laboratory examinations, the nutritional status of older adult patients with hip fractures was objectively evaluated. The susceptible patients of POD could be identified and nutritional status could be intervened preoperatively such as albumin supplementation, which provided a preliminary basis for reducing the incidence of POD. However, the current study has several limitations. Firstly, we did not measure plasma inflammatory cytokines in this study, which is a possible limitation. Secondly, this was a single-center study, and the results may not be generalizable to the general population of older adult patients. Thirdly, we used the median PNI value in POD patients as the cut-off value, and the reliability of this cut-off value requires further external validation.

## Conclusion

The current secondary analysis of two prospective, observational cohort studies demonstrated that PNI is an objective prognostic predictor of POD in older adult patients with hip fractures. Preoperative PNI combined with other risk factors, namely general anesthesia and low MMSE score, can be used as a comprehensive prognostic method of predicting POD. Perioperative management of older adult patients should involve preoperative nutritional support to decrease the incidence of POD and other complications.

## Data Availability

Data can be obtained from the corresponding author upon reasonable request.
